# Effects of Different Detraining Periods on the Physical Fitness of Older Adults with Cardiometabolic Risk Factors

**DOI:** 10.3390/ijerph21121550

**Published:** 2024-11-23

**Authors:** Mabel Diesel, Vanessa de Mattos Martini, Ian Takimoto Schmiegelow, Carlos Augusto de Sousa, Cíntia de la Rocha Freitas, Aline Mendes Gerage, Rodrigo Sudatti Delevatti

**Affiliations:** Grupo de Pesquisa em Exercício Clínico (GPEC), Centro de Desportos, Universidade Federal de Santa Catarina, R. Deputado Antônio Edu Vieira, Pantanal, Florianópolis 88040-001, Brazil; mabel.diesel@posgrad.ufsc.br (M.D.); vanessa.martini@grad.ufsc.br (V.d.M.M.); ian.takimoto@grad.ufsc.br (I.T.S.); carlos.augusto.sousa@grad.ufsc.br (C.A.d.S.); cintia.freitas@ufsc.br (C.d.l.R.F.); aline.gerage@ufsc.br (A.M.G.)

**Keywords:** older adults, detraining, cardiometabolic risk factors, physical fitness

## Abstract

Objective: To verify the effects of two different detraining periods on the physical fitness of older adults with cardiometabolic risk factors. Methods: This observational study encompassed older individuals with cardiometabolic risk factors, who were assessed after two different detraining periods: 1 month (1DT) and 3 months (3DT). Physical fitness was assessed using the 30-s sit to stand test (STS), 30-s arm curl, chair sit-and-reach, back scratch, timed up and go, and 6-min walk test (6MWT). The comparison between the different periods was carried out using absolute differences (Δ = posttest-pretest) and relative differences (Δ% = (Δ/pretest) × 100), with α = 0.05. Results: Eight older adults were assessed (70.3 ± 7.48 years, 4 female/4 male). Improvements in the STS (+1.88 repetitions; *p* = 0.007) and 6MWT (+17.38 m; *p* = 0.007) were found after 1DT. After 3DT, a worsening was observed in the 6MWT (−26.38 m; *p* = 0.018). The arm curl test worsened in both detraining periods (1DT: −1.38 repetitions; 3DT: −3.5 repetitions; *p* = 0.001). When comparing Δ% of 1DT and 3DT, STS and 6MWT showed differences, with *p* = 0.024 and *p* = 0.005, respectively. Conclusions: The 1-month detraining period had a positive effect on some physical fitness components, while 3 months induced a decline in cardiorespiratory fitness. Upper limb strength appears to be the component most susceptible to detraining.

## 1. Introduction

Population aging is an aspect of the demographic transition taking place globally, with the number of older adults aged ≥60 expected to double by 2050, and 80% of the older population living in low- and middle-income countries [1]. In this context, the World Health Organization defines healthy aging as “the process of developing and maintaining the functional ability that enables well-being in older age”, and being free of illness or disease is not a requirement for healthy aging, since many older adults have one or more health conditions which, when well-managed, have little impact on their overall well-being [2]. Maintaining physical fitness is therefore essential, as it directly influences the ability to perform daily living activities and, consequently, the health status of older adults. Lower fitness levels can make these activities more difficult, representing a risk to their independence [3].

Regarding the maintenance and improvement of physical fitness in older adults, combined training, which includes both strength and aerobic exercises, is well-supported in the literature [4,5]. Additionally, cardiorespiratory fitness plays a critical role in reducing the incidence of noncommunicable chronic diseases, overall mortality, and cardiovascular disease-related mortality [6,7], even in individuals with cardiovascular disease [8]. Low levels of muscle strength are also predictors of all-cause and cardiovascular mortality risk in adults and older adults [9,10]. Moreover, the American College of Sports Medicine guidelines emphasize the importance of aerobic exercise in maintaining and improving various aspects of cardiovascular function and health. It also enhances the ability to perform daily living activities independently, thereby improving the overall functional fitness of older adults [4].

However, older adults who participate in community programs organized by their local institutions are subject to temporary interruptions in their exercise programs due to vacation breaks [11,12,13], which can lead to the detraining effect [14]. This phenomenon, which may result in the reversibility of acquired adaptations, can occur partially or totally, depending substantially on the prior exercise stimulus and the length of activity interruption [15,16].

As numerous factors can influence responses to detraining periods, the effects of different periods of exercise cessation on physical fitness in older individuals are unclear. Most studies have evaluated only one detraining period [16,17], without direct comparisons between different periods, leading to conflicting results. For example, among healthy older adults, a short period of 4 to 6 weeks of detraining appears to reduce strength, cardiorespiratory fitness, flexibility, and agility [18,19]. On the other hand, a detraining period of 12 to 14 weeks seems to affect strength and flexibility, with conflicting results for cardiorespiratory fitness and agility/dynamic balance [11,12,20,21].

Specifically, in older adults with cardiometabolic risk factors, the evidence is even more scarce. There are indications that among older women with prehypertension, hypertension, and/or dyslipidemia, different detraining periods may cause significant decreases in different components of physical fitness, especially after 3 months of detraining [22,23]. However, when analyzing shorter periods (i.e., 4 weeks), no direct comparison between these different periods was made [23].

In summary, it appears that short detraining periods (i.e., 4 and 6 weeks) are already capable of reducing physical fitness in older adults [18,19]. However, the decline could be faster or more pronounced in those with cardiometabolic risk factors. This may be due to an inflammatory condition that affects adaptability, not allowing the most effective load increase during the training period and, consequently, the permanence of adaptations for longer periods [24,25]. Nevertheless, this association requires further investigation. Additionally, studies on older adults with cardiometabolic risk factors have focused predominantly on previously untrained older women, which makes it difficult to generalize the results to older adults of both sexes who exercise regularly.

Therefore, the aim of this study is to evaluate and compare the effects of two different detraining periods on the physical fitness of older adults with cardiometabolic risk factors who exercise regularly.

## 2. Materials and Methods

### 2.1. Study Design

This was an observational study, based on a real-life setting, and reported following the guidelines of the Strengthening the Reporting of Observational Studies in Epidemiology (STROBE). This study analyzed the database with the results of the physical/functional fitness assessments carried out at the beginning and end of two activity periods of the Cardiorespiratory Prevention and Rehabilitation Program (PROCOR).

PROCOR is a community/research program offered biannually to the community by the Federal University of Santa Catarina (UFSC) Sports Center. It aims to improve cardiovascular health and functional capacity in older individuals with cardiovascular diseases and/or cardiometabolic risk factors (hypertension, diabetes, or dyslipidemia) through supervised physical exercise provided by physical education professors and students. The health conditions of all participants were confirmed by medical certificates or prescriptions, and all participants had medical clearance to engage in physical exercise. Additionally, at the beginning of each PROCOR training period, an anamnesis was conducted to gather sociodemographic and general health information.

**Figure 1:**
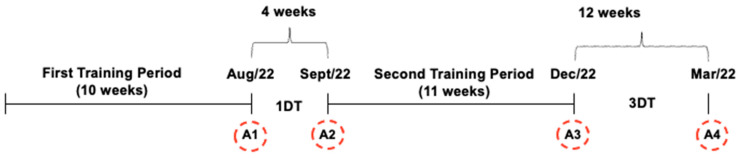
Study design; different assessments (A1, A2, A3, A4); 1 month of detraining (1DT); 3 months of detraining (3DT).

The two evaluated pause periods correspond to the mid-year and end-of-year breaks that occur in PROCOR’s community offerings. The assessments conducted at the end of the first training period and the beginning of the second training period of 2022 were used to evaluate the effects of approximately 1 month of detraining (1DT), and those conducted at the end of the second training period of 2022 and the beginning of the first training period of 2023 were used to evaluate the effects of approximately 3 months of detraining (3DT), corresponding to detraining periods of 4 and 12 weeks, respectively (Figure 1). 

### 2.2. Participants and Ethical Procedures

The study participants were older individuals who attended PROCOR activities and performed the assessments at the end of the first and the beginning of the second training period of 2022; the end of the second training period of 2022 and the beginning of the first training period of 2023. This study adopted convenience sampling, and as the inclusion criteria, participants had to attend PROCOR activities in the first and second training periods of 2022 and in the first training period of 2023. The exclusion criteria adopted were the absence of any data collected in any of the four evaluations at the beginning or at the end of the detraining periods analyzed.

As the study was carried out in a real-life setting and the data evaluated came from a database of a university community program (PROCOR), no sample calculation was carried out, as the study was observational and aimed to initially explore a dataset to possibly identify the phenomenon of detraining. 

PROCOR is approved by the Research Ethics Committee of the local university (protocol No. 3.615.659). All participants signed the Informed Consent Form at the beginning of both training periods, and the study procedures were conducted in accordance with the Declaration of Helsinki. 

### 2.3. Training Period

During the two training periods, all participants underwent a 10-week and 11-week combined training program, twice per week, respectively (Figure 1). In both, the training program started with a 1-week familiarization phase and was then divided into three 3-week mesocycles in the first training period and two 5-week mesocycles in the second training period. As PROCOR’s goal is to improve cardiovascular health and functional capacity, the training periods were periodized to progressively increase the intensity of both aerobic and strength training in the transition between the mesocycles, by reducing the number of repetitions and increasing loads in strength training, as well as increasing time spent at higher intensities in aerobic training. The training sessions lasted 50 min, consisting of 20 min of aerobic training and four strength exercises targeting the same major muscle groups, in both training periods. The combined exercise program was conducted at the UFSC Sports Center and applied by physical education students, under the supervision of postgraduate students or teachers, members specifically selected to compose the PROCOR technical staff/instructors.

### 2.4. Procedures and Outcomes 

The assessments were carried out at UFSC Sports Center facilities by physical education students, members of PROCOR’s technical staff, who were previously trained to conduct them. Six tests were performed to assess different components of functional fitness. 

#### 2.4.1. Strength

Lower limb strength was measured using the 30-s sit to stand test. A chair with a backrest and no arms was used for the test. The participant was instructed to sit on the chair without using the backrest and with their arms crossed at the chest. For safety reasons, the chair was placed against a wall. At the assessor’s signal, the participant got up, stood completely straight, and sat down again. The participant repeated this movement at their maximum speed for 30 s, and the number of times they got up from the chair was counted as one repetition. The total number of repetitions in this period was taken as the test result, and only one attempt was made at the test [26]. 

Upper limb strength was evaluated using the 30-s arm curl test, with a 2.0 kg and a 4.0 kg dumbbell for women and men, respectively. The test started with the participant sitting on a chair (same as the previous test) and positioning their arm with the elbow extended to the side of the chair and perpendicular to the floor. At the assessor’s signal, the participant flexed the elbow to full range of motion and returned the arm to the fully extended position. The participant performed as many repetitions as possible within 30 s, with only one attempt with the dominant arm. The total number of elbow flexions performed during the test (repetitions) was used as the test result [26].

#### 2.4.2. Flexibility

Lower limb flexibility was assessed using the chair sit-and-reach test. For the test, a chair with a backrest without arms and a 45 cm ruler was used. To perform the test, the participant sat down with one leg extended and slowly leaned forward with the arms extended. The distance in centimeters between the extended fingers of the hands and the toes (negative result) or the distance beyond the toes (positive result) was recorded. The test result was the highest value achieved in two attempts [26]. 

Upper limb flexibility was assessed using the back scratch test. This test measures how closely a participant can reach behind their back. Participants, in the standing position, were instructed to reach one arm over their shoulder and down their back, while reaching up with the opposite hand from behind, attempting to touch or overlap their fingers. The distance between the tips of their middle fingers was measured in centimeters. The test result was the shortest distance achieved between the extended middle fingers (negative result) or the largest overlap measure (positive result) from two attempts [26].

#### 2.4.3. Balance/Agility

Dynamic balance/agility was assessed using the timed up and go (TUG) test. The participant started the test sitting on a chair with the back against the backrest and hands crossed over the chest. At the assessor’s signal, the participant rose from the sitting position, walked 3.00 m, turned around, and returned to the seated position. The TUG was performed at two walking speeds (maximum and habitual), with two attempts for each speed, and the shortest time (seconds) for each speed was used as the test result [27]. 

#### 2.4.4. Cardiorespiratory Fitness

Aerobic capacity was measured using the 6-min walk test (6MWT). To perform the test, the participant placed themselves at the beginning of a 50-m course. At the signal from the assessor, the stopwatch was started, and the participant began to walk around the course as quickly as possible. The assessor informed the participants when there were 15 s remaining so that they could prepare for the end of the test. At the end of the six minutes, the participants stopped walking. Participants completed only one attempt, and the test result was the total distance in meters covered during this time [26].

The tests were carried out in the following order: chair sit-and-reach test, back scratch test, timed up and go (usual speed before maximum speed), 30-s sit to stand test, and 30-s arm curl test. The only test that was carried out on a separate day (for reasons of evaluation logistics) was the 6-min walk test, and the evaluations ended with its completion.

### 2.5. Statistical Analysis

The variables had their normality tested using the Shapiro–Wilk test. Variables characterized as normal are presented by mean ± standard deviation (SD), while non-normal variables are presented by median and interquartile range (IQR). Categorical variables for characterizing the sample are presented as absolute frequency (sample *n*) and relative frequency (%). Generalized estimating equation (GEE) analysis was used to compare the main effects between detraining periods, evaluating condition/group (1DT and 3DT) by time (before detraining and after detraining), adopting the Bonferroni post hoc. As the exclusion criteria adopted was the absence of any data collected in any of the four evaluations, no data were imputed. Effect sizes (ES) between groups (detraining periods) were calculated from the mean pre-post change in the 1DT minus the mean pre-post change in the 3DT, divided by the pooled pre-detraining standard deviation [28].

Additionally, to analyze the magnitude of the two detraining periods, the relative differences (Δ% = [(posttest-pretest)/pretest) × 100]) were compared. For normal variables, the student’s t-test for dependent samples was used, and the Wilcoxon test was used for nonparametric variables. The significance level adopted was 5%. The statistical analysis was carried out in SPSS (Statistical Package for the Social Sciences), version 22.0 and Rcmdr program version 4.3.1 (R commander).

## 3. Results

**Figure 2:**
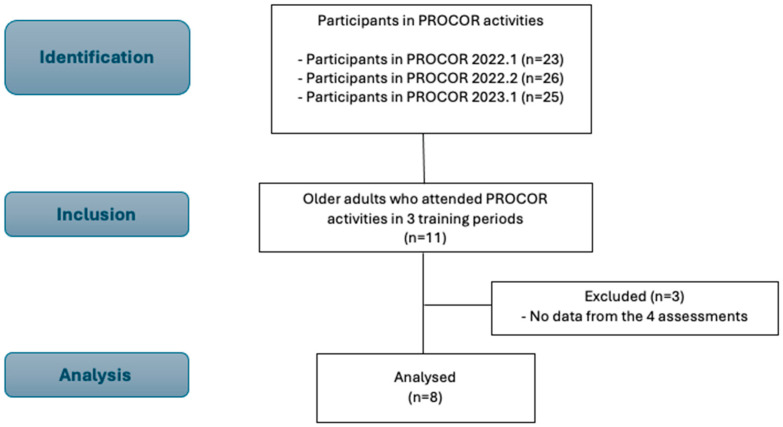
SROBE flow diagram of participants.

Eleven older adults attended all three training periods of PROCOR activities and were initially identified and included. Three participants did not perform the four assessments required to evaluate the two detraining periods and were excluded from the sample (Figure 2). Thus, eight older adults participated in the study, 50% female and 50% male. In general, the participants were overweight, and the most prevalent cardiometabolic risk factors were hypertension and dyslipidemia (Table 1).

Table 2 presents the results of the two detraining periods. A statistically significant improvement was identified for lower limb strength (+1.88 repetitions; *p* = 0.007) and cardiorespiratory fitness (+17.38 m; *p* = 0.007) after 1DT. Regarding 3DT, a significant reduction was identified for cardiorespiratory fitness (−26.38 m; *p* = 0.018). Effect sizes between detraining periods were considered medium for cardiorespiratory fitness and large for lower limb muscle strength. Upper limb strength was the only physical fitness component that decreased during both detraining periods (1DT: −1.38 repetitions; 3DT: −3.5 repetitions; *p* = 0.001) with small effect size.

A statistically significant difference was also found between 1DT and 3DT for lower limb strength before detraining (3.00 repetitions, *p* < 0.001) and for cardiorespiratory fitness after detraining (30.25 m; *p* = 0.003). For the other physical fitness components, no significant differences were observed (all *p* > 0.05).

When comparing the percentage changes in the different physical fitness components between the detraining periods, the results show that the Δ% had a different pattern for cardiorespiratory fitness and lower limb strength, with improvements observed only during the short detraining period (Table 3).

## 4. Discussion

This study assessed the impact of two distinct detraining periods—short (4 weeks) and long (12 weeks)—on six components of health-related physical fitness in older adults with cardiometabolic risk factors. Contrary to initial expectations, the results showed that detraining did not cause significant losses in most of the assessed outcomes, even after a 3-month period. The main finding was that distinct responses occurred in the short and long detraining periods, with improvements observed in cardiorespiratory fitness and lower limb strength after 1 month, while the 3-month period resulted in a loss of cardiorespiratory fitness. Upper limb strength was the only component that showed losses in both detraining periods.

Studies that examined short detraining periods following multicomponent training programs (including combined training) demonstrated overall declines in physical fitness over a 6-week period [19] or partial declines over a 4-week period [23] in healthy and prehypertensive older adults, respectively. Other studies that solely conducted strength training observed similar results over a 4-week detraining period in older adults [18,29]. The discordant response found in this study, particularly regarding the improvements in lower limb strength and cardiorespiratory fitness, could potentially be attributed to a process of supercompensation. This phenomenon occurs when the body adapts and improves in response to the stress of prior training [30]. As the periodization used in both training periods is based on progressive intensity, with the highest intensity of exercise in the final mesocycle, this hypothesis is plausible for explaining the observed result. Higher intensity mesocycles need to be followed by longer rest or by a regenerative mesocycle so that supercompensation can occur, helping the individual adapt physiologically to the previous stimuli.

Another point to highlight is that the observed improvement in lower limb muscle strength may have influenced cardiorespiratory capacity, as achieving satisfactory cardiorespiratory levels requires that the lower limbs be fit for such, in addition to the necessary pulmonary and cardiac physiological adaptations [31]. Indeed, the literature has indicated that 20 weeks of strength training can improve the 6MWT, reinforcing the influence of lower limb strength on better cardiorespiratory performance [29]. We would also like to mention that both tests that showed improvements in 1DT represent the most important physical fitness components for carrying out daily living activities, therefore having an impact on the preservation of physical independence in older adults [4].

During the 4-week detraining period, in addition to the mentioned improvements, no significant changes were observed in flexibility or agility/dynamic balance. This finding contrasts with the results of Silva et al. [25], who identified a decline in flexibility after 1 month of detraining in older adults with metabolic syndrome. This difference in results may be related to the specificity principle in fitness, which states that training adaptations are specific to the applied stimulus and the specificity of the exercise program. When flexibility is not trained (as in the case of PROCOR), this component of physical fitness does not undergo significant changes during the training period and consequently did not show significant changes during the detraining period in older adults, especially in the upper limbs [29,32].

The explanation above may also apply to the lack of results for flexibility in the 3DT and agility/dynamic balance in both detraining periods [33]. Additionally, a cut-off of 12 s for the TUG is associated with negative outcomes for older adults [34,35], with this time being much longer than that found after 3DT, thus highlighting the high physical condition of the participants in this study in this aspect, reducing the margins for its trainability, as well as for its detraining capacity. 

After both detraining periods, a significant reduction in upper limb strength was observed, with a small effect size between 1DT and 3DT. This result is partially in line with other studies that found reductions in different components of physical fitness, including upper limb strength, after a 4-week detraining period for older women who participated in a strength training program [18,29]. On the other hand, when analyzing 4 and 12 weeks after multicomponent training in prehypertensive older women, there was no decrease in upper limb strength, which could possibly be explained by the different assessment method used, which was handgrip [23]. The result of decreased upper limb strength found here may be related to the lesser use of the upper limbs in daily activities compared to the lower limbs.

Cardiorespiratory fitness is the other component that presented a reduction after 3DT. In general, other studies found negative effects on different physical fitness components with 3 months of detraining following multicomponent training programs in older women with cardiometabolic risk factors [21,22,23], and the reduction in cardiorespiratory fitness after 3 months of detraining seems to be an aspect they have in common, which corroborates the results presented here. On the other hand, it is important to note that healthy older adults do not appear to experience significant losses in this capacity after 3 months of detraining from a multicomponent training program [12,20].

When comparing 1DT and 3DT (relative differences), differences in behavior for cardiorespiratory fitness and lower limb strength were found, thus corroborating the analyses previously made, that these two components improved during the 1-month detraining period and were lost and maintained, respectively, during the 3-month period. The medium effect size found for cardiorespiratory fitness and the large effect size found for lower limb strength between the detraining periods reflect the difference between the Δ % for these components (7.6 percentage points for the 6MWT and 20.6 percentage points for the sit and stand test).

The maintenance of physical qualities after both detraining periods, but especially in the 3DT, may be related to the experience and “training status” of the participants, since the 8 older adults analyzed have been part of PROCOR for at least 10 years. Studies indicate that the total training time plays a crucial role in maintaining exercise adaptations and long-term improvements. Esain et al. [12] evaluated older adults with 12 years of multicomponent training practice and found no differences after 3 months of detraining, except for the TUG. Douda et al. [13] observed that, although 3 months of detraining reversed favorable adaptations obtained during 9 months of training, after 5 years, the adaptive response led to significant improvements in older women. The findings highlight the importance of the continuity of physical exercise programs for older adults over the long term, helping to preserve and enhance functional fitness and reduce health risks and loss of independence.

Additionally, many participants reported remaining physically active during both detraining periods, which might have contributed to maintaining the outcomes, since five of the eight evaluated participants maintained regular physical activities, such as walking and stretching during the breaks. Martínez-Aldao et al. [36] demonstrated that older adults who remain more physically active during detraining periods are likely to experience lower losses in different components of physical fitness over a 5-month period. Therefore, it can be speculated that if these same older adults had been analyzed over a shorter detraining period, they might have shown results similar to those found in this study. In addition, we believe that the practice of physical activity may have played the role of a potential regenerative mesocycle in 1DT, promoting, to some extent, the process of supercompensation.

We would also like to highlight that the results found in this study are possibly due to the specificity and intensity progression used in both training periods, combined with the adherence of the participants, which was around 72%. Lastly, although we could not find statistically significant differences between 1DT and 3DT for the other outcomes, when observing 3DT, we noticed a worsening pattern (or a reduction of greater magnitude compared to 1DT) in all components of physical fitness assessed, possibly indicating the beginning of a generalized loss.

This study presents some limitations. Firstly, the lack of a control group and blinding of assessors, which would not be possible due to the nature of the community program from which the data originated. On the other hand, we believe that the presence of a comparator group (which remains training during periods of detraining) would be the ideal design for future research. The small sample size also becomes a limiting factor, as it may have interfered with the detection of significant changes in other components of physical fitness. Future studies comparing different periods of detraining should be carried out with a larger number of participants to detect differences with greater certainty. The lack of a specific and precise instrument to assess physical activity levels also becomes a limiting factor, as it is a variable that can directly interfere with the results found. However, despite the limited data (we did not assess the level of physical activity but only its practice or not), the participants who reported engaging in physical activity in both periods were the same, so we believe that this influence can be minimized by this factor.

However, we highlight that there are few studies that assess detraining in older individuals with cardiometabolic risk factors and compare different detraining periods [22,23]. To our knowledge, this is the first study to address detraining in older adults of both sexes with these clinical characteristics and who exercise regularly. Although the study included a small number of participants, it provides preliminary results of a behavior that is possibly repeated over the years, which will be investigated in the future by our research group. Additionally, several studies that have evaluated detraining and older adults do not provide detailed information about the presence of cardiometabolic risk factors or cardiovascular diseases in the participants [11,12,19,29], which are possibly present, and this may influence the divergence of results found in the literature to date.

## 5. Conclusions

This study has shown that detraining periods of 4 and 12 weeks can have different impacts on the physical fitness of older adults with cardiometabolic risk factors. A 1-month break resulted in improvements in cardiorespiratory fitness and lower limb muscle strength, possibly due to a phenomenon of metabolic supercompensation, and a 3-month break did not have a negative impact on most components of physical fitness, compromising only upper limb strength and cardiorespiratory fitness. Upper limb strength was the only decreased component in both detraining periods. It is possible that interruptions longer than 3 months could lead to impairment of the physical adaptations previously achieved. We emphasize that the findings need to be interpreted with some caution, as they come from preliminary results and a small sample size.

## Figures and Tables

**Table 1:** Characteristics of participants (*n* = 8).

Characteristics	
Sex (Male/Female)	4/4
Age (years)	70.3 ± 7.48
BMI (kg/m^2^)	28.6 ± 4.18
BMI 18.5–24.9, n (%)	1 (12.5%)
BMI 25–29.9, n (%)	4 (50%)
BMI > 30, n (%)	3 (37.5%)
Hypertension, n (%)	6 (75%)
Diabetes mellitus, n (%)	3 (37.5%)
Dyslipidemia, n (%)	6 (75%)

Data described by mean ± standard deviation, absolute and relative frequency (%); BMI: Body mass index.

**Table 2:** Effects of different detraining periods on participants’ physical fitness (*n* = 8).

Outcomes	4 Weeks of Detraining (1DT)		12 Weeks of Detraining (3DT)		*p*-Value			
	Before Detraining	After Detraining	Before Detraining	After Detraining	Group	Time	Group × Time	ES
6MWT (m)	572.70 ± 70.02	589.87 ± 77.2 ^#^*	586.00 ± 77.04	559.62 ± 89.10 ^#^*	0.265	0.562	<0.001	0.56
Arm Curl (rep)	21.12 ± 5.19	19.75 ± 3.88	22.50 ± 5.37	19.00 ± 4.65	0.752	0.001	0.158	0.38
Sit to Stand (rep)	14.25 ± 2.25 ^#^	16.12 ± 3.87 *	17.25 ± 3.15 ^#^	15.63 ± 2.66	<0.001	0.802	0.003	1.21
Back Scratch (cm)	−7.37 ± 14.45	−9.18 ± 16.73	−6.81 ± 14.44	−6.93 ± 14.18	0.041	0.231	0.373	−0.11
CSR (cm)	−2.93 ± 14.62	0.06 ± 9.46	−1.25 ± 11.92	−1.12 ± 8.77	0.832	0.367	0.208	0.20
TUG_Max (s)	5.91 ± 1.29	6.03 ± 1.00	6.10 ± 1.03	6.27 ± 0.96	0.162	0.353	0.866	−0.04
TUG_Usual (s)	7.29 ± 0.90	7.30 ± 0.62	7.32 ± 1.12	7.70 ± 0.76	0.186	0.412	0.059	−0.34

Data described by mean ± standard deviation; 6MWT: 6-min walk test; CSR: Chair sit-and-reach test; TUG: Timed up and go test; m: Meters; rep: Repetitions; s: Seconds; cm: Centimeters; ^#^: Difference between groups (*p* < 0.05); *: Different from baseline (*p* < 0.05); *p*-values were determined by generalized estimating equation.

**Table 3:** Comparison between different periods of detraining on the physical fitness of participants (*n* = 8).

Outcomes	1DT (Δ%)	3DT (Δ%)	*p*-Value
6MWT (%)	2.99 ±3.40	−4.65 ± 6.38	0.005
Arm Curl (%)	−11.90 (19.31)	−15.07 (15.72)	0.382
Sit to Stand (%)	12.20 ±13.48	−8.38 ± 14.86	0.024
Back Scratch (%)	6.60 ± 90.67	0.07 ± 37.18	0.832
Chair Sit-and-Reach (%)	16.29 (53.80)	4.54 (55.54)	0.312
TUG_Max (%)	0.90 (9.03)	1.43 (17.68)	0.640
TUG_Usual (%)	0.89 ±9.20	6.38 ±12.47	0.091

Data described by mean ± standard deviation, median (IQR); 1DT: 4 weeks of detraining; 3DT: 12 weeks of detraining; 6MWT: 6-min walk test; TUG: Timed up and go test; Δ% = [(posttest-pretest)/pretest) × 100]; *p*-values were determined by student’s t-test for dependent samples or Wilcoxon test.

## Data Availability

Available through the corresponding author by request.
